# Social capital and dietary patterns in three ethnic minority groups native to Yunnan Province, Southwest China

**DOI:** 10.1371/journal.pone.0256078

**Published:** 2021-08-12

**Authors:** Qiang Zhang, Zhitao Liu, Wenmin Hu, Xinguang Chen, Juanjuan Li, Qingqing Wan, Jiang Zhao, Yuan Ruan, Baoqing Dao, Yunfei Li, Xiangdong Min

**Affiliations:** 1 Department of Nutrition and Food Hygiene, Yunnan Center for Disease Control and Prevention, Kunming, China; 2 Department of Epidemiology, University of Florida, Gainesville, Florida, United States of America; 3 Department of Public Health, Dehong Center for Disease Control and Prevention, Mangshi, China; 4 Department of Health Education, Lanping Center for Disease Control and Prevention, Lanping, China; University of Western Australia, AUSTRALIA

## Abstract

**Background:**

Few studies have focused on the influencing factors of dietary practices among ethnic minority groups in China, particularly from a social capital perspective.

**Methods:**

Between May and September 2019, we conducted a cross-sectional survey among adults (n = 1,813) from three ethnic minority communities (A Chang, De Ang and Jing Po) in Yunnan Province, Southwest China. Dietary intakes during the past 12 months were measured with a 100-item Food Frequency Questionnaire (FFQ), and two forms of social capital (bonding and bridging) were measured using the validated Personal Social Capital Scale 16 (PSCS-16). Principal component factor analysis was used to derive dietary patterns from 20 food groups. Multivariate linear regressions were used to examine the associations between social capital and dietary patterns.

**Results:**

Two distinct dietary patterns were identified: the traditional and the modern. The traditional pattern was characterized by high consumptions of tubers, poultry, rice, fruits, vegetables and low consumptions of oil and salt, whereas the modern pattern was highly correlated with egg, nut, beverage, snack and oil consumptions. After adjusted for potential confounders, the modern pattern was positively associated with bonding capital (*β* = 0.066; 95%CI: 0.058, 0.075) and negatively associated with bridging capital (*β* = -0.017; 95%CI: -0.024, -0.010).

**Conclusion:**

In conclusion, an unhealthy dietary pattern was identified among the ethnic minority groups in Southwest China. The influences of people’s social connections on dietary behaviors should be considered in designing and implementing nutrition intervention programs for the population.

## Introduction

Good nutrition is vital for both physical and mental health [1]. However, it is estimated that 22% of global deaths among adults in 2017 were attributable to dietary risk factors [2]. During the past decades, China has also experienced a rapid nutrition transition characterized by unhealthy changes in eating behaviors and a surge in non-communicable diseases, even in less developed areas [3–5]. Given the challenges above, more in-depth researches on factors that affect diet are urgently needed.

Apart from factors such as age, sex, education and income, the influence of social capital on dietary behaviors have received increasing attention in recent years. Social capital can be conceptualized at both the collective and individual levels, e.g., either as the features of social organization, or as the personal resource emerges from social network [6, 7]. Social capital has been recognized as a health determinant, while health-related behavior is proposed to be a mechanism [8, 9]. Studies from China and abroad showed that people having high individual social capital are more likely to adhere to a healthy diet [10, 11]. In this sense, social capital intervention might be a promising approach to promote healthy eating. However, individual social capital is also a multifaceted concept [12]. According to social networks and the interactions within, social capital can be divided into bonding, bridging and linking capital. Bonding capital refers to “inward” connections between homogeneous individuals, bridging capital refers to “outward” connections between heterogeneous individuals, and linking capital characterizes connections between individuals across authority gradients [13, 14]. Because of this, different types of social capital may have different effects on individual dietary practices. A study from Spain indicated that family and peers are the most influent sources of social capital in relation to adolescent dietary behaviors, including both protective and damaging effects [15]. Moreover, a recent study on older adults in China showed that people with higher bonding and bridging social capital are less likely to skip breakfast [16]. Thus, studies that assess the effects of types of social capital on overall diet quality are needed.

Dietary pattern analysis is a holistic and data-driven approach to evaluate dietary behaviors [17]. By assessing dietary intakes of individuals as a whole, dietary patterns can reflect the combined effects of multiple foods and nutrients on health outcomes [18, 19]. Moreover, dietary patterns are also strongly correlated with socioeconomic characteristics, which is helpful to determine dietary preference across subgroups [19]. To date, two main dietary patterns (the traditional and the modern) have been identified in Chinese adult population [20, 21]. The traditional pattern is characterized by high intakes of rice, wheat and vegetables, which represents a typical traditional diet in China. The modern pattern is characterized by high intakes of meat, milk and fast foods, and often associated with higher risk of obesity, diabetes and hypertension. Longitudinal studies using the China Health and Nutrition Survey (CHNS) data reported dramatic changes in dietary patterns, reflecting the shift from the traditional agricultural society to a modern commercial and industrialization society [22, 23]. By contrast, dietary patterns among Chinese ethnic minority groups are less clear and understudied. With a focus on disease prevention and health promotion, Healthy China Initiative (2019–2030) has proposed 15 special campaigns including a balanced diet action [24]. This action again emphasizes the need to implement dietary guidance and nutrition intervention, as well as to provide community nutrition instructors. Yet, physical and human resources for health are often scarce, especially for ethnic minorities living in rural areas. As a type of important resource, social capital should be considered when planning nutrition promotion interventions.

There are 15 ethnic minority groups native to Yunnan Province, Southwest China. Most of them live in areas with a backward economy and poor public transportation. Benefited from China’s targeted poverty alleviation policy in recent years, the local economy has developed at a high speed, which would also lead to dramatic changes in social connections and diets. Therefore, we hypothesize that bonding and bridging social capital may have different roles on the nutrition transition in this context. The purpose of this study was to identify and characterize dietary patterns, as well as to examine their associations with social capital in the ethnic minority groups.

## Materials and methods

### Study design

Between May and September 2019, we conducted a cross-sectional survey among adults of three ethnic minority groups in Yunnan Province, which were A Chang, De Ang and Jing Po. According to the sixth national census in 2010, total population for each of the minorities were less than 150,000 and most of them live in certain border towns [25]. Participants were recruited using a multi-stage sampling method. First, three towns had the largest population of A Chang, De Ang or Jing Po were chosen as the study sites. Then, two villages were randomly selected from each town with the Probability Proportional to Size method. Finally, based on local household registration information, 150 households were randomly selected from each village. All adults of the sampled households were invited to participate the survey, except those who were on a prescribed diet or who had serious illnesses.

The sample size was determined from careful power analysis considering the following four factors: a prevalence of hypertension of 15%, a desired precision of 3%, a type I error of 0.05 and the population of each ethnic minority group. Since hypertension is the most prevalent nutrition-related disease in the ethnic minorities, the prevalence we determined in a prior study was used to calculate the sample size [26]. The minimum sample size per minority group ranged from 538 to 554. We aimed at recruiting 580 participants per site, considering potential of 5% non-response. Data were collected through a face-to-face interview by trained local health workers. Altogether, 1813 participants completed the survey with a response rate of 85%. The study was approved by the Institutional Review Board at Yunnan Center for Disease Control and Prevention (2019–01). Before administrating the survey, signed informed consent was obtained from all the participants.

### Dietary assessment and dietary patterns

Dietary intakes in the past 12 months were assessed using the Food Frequency Questionnaire (FFQ) developed by the Institution of Nutrition and Health, Chinese Centers for Disease Control and Prevention, which has been widely used in previous national nutrition survey [27]. The FFQ consists of 100 detailed food items, covering most of the commonly consumed foods in China. Based on the frequency and amount of food intakes reported by individual participants, daily intake of each food item was calculated. Due to the low intake of some food items, these 100 detailed food items were then grouped into 20 food groups according to the China Food Composition Table for further analysis [28]. These food groups were rice, wheat, tubers, beans, vegetables, salt vegetable, mushrooms, fruits, milk, red meat, poultry, organ meat, fish, eggs, cakes, nuts, snacks, beverages, salt and oil.

Dietary patterns were identified using factor (principal component) analysis based on the daily consumptions of the 20 food groups. Principal component analysis is a data driven technique that reduces the dimension of the data and groups correlated variables, to form new components. The number of factors retained was determined by eigenvalue (>1.0), scree plot, factor interpretability, and the variance explained (>5%). The factors were rotated by an orthogonal rotation to achieve a simpler structure with greater interpretability. Factor loadings, which presenting the correlations between food groups and factors, were calculated for each food group across the two factors. Higher loadings indicate a higher shared variance with the factor. Factor loadings of > |0.25| represent the food groups that most strongly related to the identified factor. Dietary patterns were named according to the food groups showing high loadings for each factor. Pattern-specific scores were then calculated and assigned to each participant. Factor scores for each pattern were categorized into quartiles (quartiles 1 and 4 representing low and high adherence to each dietary pattern, respectively).

### Measurements of social capital

Social capital for individual participants was assessed using the Personal Social Capital Scale 16 (PSCS-16). This scale consists of 16 items with 8 items measuring the bonding capital (including network, trust, resource ownership and reciprocity) and eight items measuring bridging capital, embedded in two types of social organizations (political and economic organizations; recreational and cultural organizations). Reponses for each item were ranked on a five-point Likert scale. Mean scores for the bridging and bonding capital were calculated and used in the following descriptive and regression analysis. In previous studies in China, Cronbach’s α of the scale ranged from 0.81 to 0.87 [29, 30]. Correlation analysis showed that the alpha coefficient in this study was 0.87. The PSCS-16 can be found in S1 Table.

### Covariates

Sociodemographic characteristics of the participants were incorporated in the analyses as potential confounders, including age, sex, ethnicity, education and income. Age was divided into five groups (18–34, 35–44, 45–54, 55–64 and ≥65 years) in the descriptive statistics, while treated as a continuous variable in the regression analyses. Education was classified as three levels (illiteracy, primary school, middle school or higher). Income was measured by household income per capita in the last 12 months and divided into four categories (<5000, 5000–9999, 10000–14999, >15000 Yuan).

### Data analysis

Correlation analyses were used to calculate Cronbach’s α of the social capital scale. Categorical variables (e.g., age and sex) are presented as percentage, while variables (e.g., social capital scores) were presented as mean and standard deviation (SD). Chi-square tests were used to examine the trend in sociodemographic characteristics across the quartiles of dietary patterns. Linear regression analyses were used to estimate the associations between social capital and dietary pattern scores with potential confounders adjusted. All statistical analyses were performed with SAS Software 9.4 (SAS Institute, Cary, North Carolina, USA). Statistical significance was set at *P*< 0.05.

## Results

Sociodemographic characteristics of the participants are presented in Table 1. The total sample included 745 men and 1068 women. The proportions of participants aged 18–34, 35–44, 45–54, 55–64, and 65 years or older were 16.7%, 21.1%, 24.0%, 25.2% and 12.9%, respectively. Each of the three ethnic minority group accounted for one third of the sample. Nearly 65% of the participants had an annual income less than 10,000 RMB Yuan (equivalent to 1,400 US dollars), and over 80% only had primary education or no formal education.

**Table 1:** Sociodemographic characteristics of the participants.

Characteristics	Men	Women	Total
Sex, N (%)	745 (41.1)	1068 (58.9)	1813 (100.0)
Age in years, N (%)			
18–34	155 (8.5)	148 (8.2)	303 (16.7)
35–44	182 (10.0)	201 (11.1)	383 (21.1)
45–54	153 (8.4)	282 (15.6)	435 (24.0)
55–64	155 (8.5)	302 (16.7)	457 (25.2)
≥65	100 (5.5)	135 (7.4)	235 (12.9)
Ethnicity, N (%)			
A Chang	233 (12.9)	377 (20.8)	610 (33.6)
De Ang	291 (16.1)	320 (17.7)	611 (33.7)
Jing Po	221 (12.2)	371 (20.5)	592 (32.7)
Education, N (%)			
Illiteracy	143 (7.9)	466 (25.7)	609 (33.6)
Primary school	419 (23.1)	440 (24.3)	859 (47.4)
Middle school or higher	183 (10.1)	162 (8.9)	345 (19.0)
Income (Yuan/Year), N (%)			
<5000	192 (10.6)	253 (14.0)	445 (24.5)
5000–9999	267 (14.7)	438 (24.2)	705 (38.9)
10000–14999	148 (8.2)	203 (11.2)	351 (19.4)
≥15000	138 (7.6)	174 (9.6)	312 (17.2)

Table 2 summarizes the two dietary patterns among the participants. The first one was labeled as the traditional pattern, which characterized by high intakes of tubers, poultry, rice, fruits, vegetables and low intakes of oil and salt. The other one which highly correlated with egg, nut, beverage and snack intakes, was named as the modern pattern. The two dietary patterns explained 28.3% of the total variance in dietary consumptions.

**Table 2:** Factor loadings of the food groups in the two dietary patterns.

Traditional	Factor loadings	Modern	Factor loadings
Tubers	0.68	Eggs	0.65
Poultry	0.68	Nuts	0.64
Fruits	0.66	Beverages	0.54
Rice	0.53	Snacks	0.50
Vegetables	0.53	Oil	0.43
Mushrooms	0.52	Cakes	0.42
Fish	0.49	Organ meat	0.40
Beans	0.40	Red meat	0.37
Salt vegetables	0.29	Salt	0.34
Salt	-0.26	Vegetables	-0.27
Oil	-0.25		
Variance explained (%)	18.7		9.6

Note: Absolute value <0.25 are not presented in the table for simplicity.

Table 3 shows the sociodemographic characteristics of the participants across quartiles of the dietary patterns. People with lower income (<10000 Yuan per year) were more likely to consume the traditional diets. In contrast, people who preferred the modern pattern were younger (<45 years), had higher educational levels (primary school or higher) and income (≥10000 Yuan per year).

**Table 3:** Sociodemographic characteristics of the participants across quartiles of the dietary patterns.

	Dietary Pattern Quartiles				*P* for trend
	Q1	Q2	Q3	Q4	
N	454	453	453	453	
Traditional					
Men (%)	39.7	39.5	41.5	43.7	0.17
Age <45 years (%)	30.4	42.8	34.4	43.7	0.13
Illiteracy (%)	34.1	35.1	21.1	34.0	0.66
Income <10000 Yuan (%)	55.7	63.6	64.5	69.9	<0.01
Modern					
Men (%)	37.0	42.2	40.8	44.4	0.06
Age <45 years (%)	29.3	39.1	40.2	42.8	<0.01
Illiteracy (%)	48.0	33.1	30.2	23.0	<0.01
Income <10000 Yuan (%)	79.1	70.6	63.8	40.2	<0.01

Table 4 presents bonding and bridging social capital scores by sociodemographic characteristics. Mean scores of bonding and bridging social capital were 21.3 and 19.9, respectively. Bonding social capital scores were higher among men and those who were younger, had higher income or education levels. No such associations were observed for the bridging capital scores.

**Table 4:** Social capital scores by sociodemographic characteristics.

	Bonding capital Mean (SD)	Bridging capital Mean (SD)
All	21.3 (5.3)	19.9 (5.7)
Sex		
Men	21.8 (5.4)	19.8 (5.8)
Women	20.9 (5.2)	20.0 (5.7)
*P*	<0.01	0.50
Age in years		
18–34	22.4 (5.1)	19.8 (5.6)
35–44	22.1 (5.2)	20.1 (5.8)
45–54	21.3 (5.5)	20.2 (5.9)
55–64	21.0 (5.2)	20.0 (5.8)
≥65	19.4 (5.0)	19.1 (5.5)
*P*	<0.01	0.18
Education		
Illiteracy	19.1 (5.8)	20.1 (5.6)
Primary school	22.0 (5.2)	19.3 (5.8)
Middle school or higher	23.6 (5.0)	20.4 (5.8)
*P*	<0.01	<0.01
Income (RMB Yuan)		
<5000	20.1 (5.6)	19.5 (5.9)
5000–9999	20.5 (5.4)	20.6 (5.9)
10000–14999	21.7 (5.8)	20.1 (5.9)
≥15000	24.4 (5.9)	18.8 (5.6)
*P*	<0.01	<0.01

Associations of dietary pattern scores with bonding and bridging capital are presented in Table 5. After adjustment for sex, age, education, income and ethnicity, the traditional dietary pattern scores were negatively associated with bonding capital (β = -0.029; 95%CI: -0.039, -0.019), while the modern dietary pattern scores were positively associated with bonding capital (β = 0.066; 95%CI: 0.058, 0.075) and negatively associated with bridging capital (β = -0.017; 95%CI: -0.024, -0.010).

**Table 5:** Associations of dietary pattern scores with social capital.

Dietary patterns	Bonding		Bridging	
	β (95% CI)	SE	β (95% CI)	SE
Traditional				
Model 1	-0.028 (-0.037, -0.020) *	0.004	-0.004 (-0.012, 0.004)	0.004
Model 2	-0.029 (-0.039, -0.019) *	0.005	-0.005(-0.013, 0.004)	0.004
Modern				
Model 1	0.083 (0.075, 0.091) *	0.004	-0.022 (-0.030, -0.015) *	0.004
Model 2	0.066 (0.058, 0.075) *	0.004	-0.017 (-0.024, -0.010) *	0.003

Model 1: Crude model; Model 2: Adjustment for sex, age, education, income and ethnicity; CI: Confidence Interval, SE: Standard Error

*P < 0.05.

## Discussion

The cross-sectional study examined the associations between dietary patterns and two types of individual social capital among ethnic minority adults in Southwest China. Two distinct dietary patterns were identified: the traditional (healthy) and the modern (unhealthy). We found that the modern pattern was positively correlated with bonding capital but negatively correlated with bridging capital. Our findings highlight the potential roles of social capital on healthy eating. To our best knowledge, this study is one of the first to assess the influence of social capital on dietary patterns among ethnic minority groups in China.

Traditional Chinese diets usually consist of a carbohydrate staple (e.g., wheat in the north and rice in the south), moderate amounts of animal-source foods, beans and a lot of vegetables [31, 32]. Because of this, it is considered to be well-balanced, rich in fiber and low in saturated fatty acids [33]. Longitudinal studies show that the traditional Chinese dietary pattern is negatively associated with weight gain [23, 34]. The traditional pattern we identified in this study shares large features with those in previous studies, except for the high loadings for poultry and fruits. It’s probably because these ethnic minority groups live in sub tropic mountain areas where abounds with fruits and family poultry faming is also very common there. By contrast, the modern dietary pattern (also known as western pattern) is an energy-dense diet, characterized by a high intake of eggs, meats, sweets and fast foods. A robust and positive association between modern pattern and obesity has so far been established both in developed and developing countries, and its other health risks are gradually recognized [35, 36]. Thus, identifying dietary patterns in a given population can provide guidance for nutrition intervention and education. Consistent with previous studies, we found that the modern pattern is more prevalent among younger people and those who have higher income and education [20, 37]. This finding emphasizes the need to pay more attention to these subgroups in the future. In general, the dietary pattern analysis reveals the challenges of unhealthy eating among the ethnic minority groups in Southwest China.

Another important finding of this study is that the modern dietary pattern is positively associated with bonding capital. This result is something different from previous studies. In these studies, people with higher individual social capital are more likely to have interest in healthy eating, consume more vegetables and fruits or adhere to a Mediterranean diet [38, 39]. This discrepancy may be due to specific social contexts in different studies. At individual level, social capital can be defined as the sum of resources that are available in one’s social networks. Social capital may influence health-related behaviors through social norms, values and attitudes prevalent in the networks [13, 40]. That is to say, the effects of social capital on dietary behaviors are largely determined by how most network members perceive healthy eating [41]. In developed countries, people usually have relatively high knowledge and awareness about good nutrition [42]. As a result, social capital in different networks always show protective effects on healthy eating. In contrast, life of the ethnic minority groups in the study has just transited from a subsistence level to a well-level. In this case, both the participants and the homogenous individuals (e.g., their family and friends) would care more about “eat satisfied” instead of “eat healthy”. This might explain why bonding social capital is related with unhealthy dietary pattern in this study. A similar finding also appears in a child nutrition study from India [43]. Therefore, the influences of bonding social capital on dietary behaviors should be evaluated in different social contexts.

Moreover, our finding that bridging social capital is a protective factor for healthy eating is consistent with existing literatures. Those studies show that participating in social organizations (the process to build bridging social capital) is correlated with better self-rated health, mental health and protective behaviors [44–46]. According to social capital theory, bridging social capital allows for the generation of ideas and innovations that would be unlikely to manifest among like-minded individuals such as family members and close friends [47]. This is especially important for healthy eating promotion among the public, for which the spread of updated and accurate nutrition knowledge is essential. From this point of view, bridging social capital may promote healthy eating by improving the access of external health information and services [48]. A recent community-based randomized trial in Nepal shows that nutrition education combined with social capital development has better effects on diet quality and growth among children than nutrition education alone [49]. Furthermore, the relationships between bridging social capital and dietary intakes may be more impactful in socioeconomic disadvantaged populations. For example, a study from Netherland shows that having bridging social capital reduce the likelihood to report overweight and obesity in low educated adults [14]. A possible explanation is that health information and services within the social network is indispensable for the low educated to prevent weight gain, but not for the educated ones. In this study, bridging social capital scores are insignificant across sex or age groups, which is inconsistent with previous studies [50]. This probably because the male young adults usually migrate to work for most of the year, which may reduce their participation in local organizations. Thus, for the ethnic minority groups living in less developed areas of Southwest China, influences of local organizations especially from ethnic cultural organizations should be fully recognized and cultivated when designing nutrition intervention programs.

Several limitations of the study should be addressed. First, data used for this study are cross-sectional, thus causal inferences cannot be warranted. Second, the dietary assessments are based on food consumption in the past 12 months, and recall bias cannot be eliminated. Third, although socioeconomic factors are adjusted in the regression models, potential factors at contextual levels for both diets and social capital are not fully considered. Last but not least, we only measured bonding and bridging social capital in this study. Additional studies are needed to examine social capital in more detail, including their relationships with dietary practices. Despite the limitations, findings of this study provide useful data for researchers to understand the dietary behaviors of ethnic minority groups in Southwest China and their relations with social capital.

## Conclusions

In conclusion, a modern dietary pattern is identified among the ethnic minority groups in Southwest China, which may be attributable to the urbanization in recent years. Modern patterns characterized by high consumptions of animal-source foods, oil and beverages have been proved to be correlated with increased risk of non-communicable diseases in the literature. In this study, we find that the modern pattern is positively associated with bonding social capital but negatively associated with bridging social capital. Our findings highlight the need to further understand the roles of different social capital on dietary behaviors when designing nutrition interventions.

## Supporting information

S1 Table Personal Social Capital Scale 16 (PSCS-16). (PDF)

S1 Data (XLS)
