# Supplementary material for: Social capital and dietary patterns in three ethnic minority groups native to Yunnan Province, Southwest China
Source: PLoS One. 2021 Aug 12;16(8):e0256078. doi: 10.1371/journal.pone.0256078 (PMC8360576; doi:10.1371/journal.pone.0256078)
Supplement: S1 Table — (PDF) [file pone.0256078.s001.pdf]

Personal Social Capital Scale 16

1. How do you rate the number of your friends?
   ①A few ②Less the average ③Average ④More than average ⑤A lot
2. How do you rate the number of your country fellows/old classmates?
   ①A few ②Less the average ③Average ④More than average ⑤A lot
3. Among your friends and classmates, how many can you trust?
   ①A few ②Less the average ③Average ④More than average ⑤A lot
4. Among your relatives, how many can you trust?
   ①A few ②Less the average ③Average ④More than average ⑤A lot
5. Among all your relatives, neighbors, friends, co-workers, and classmates, how many have broad connections with others?
   ①A few ②Less the average ③Average ④More than average ⑤A lot
6. Among all your family members, relatives, neighbors, friends, co-workers, and old classmates, how many are with a professional job?
   ①A few ②Less the average ③Average ④More than average ⑤A lot
7. How many of your coworkers/fellows will definitely help you upon your request?
   ①A few ②Less the average ③Average ④More than average ⑤A lot
8. How many of your friends will definitely help you upon your request?
   ①A few ②Less the average ③Average ④More than average ⑤A lot
9. How do you rate the number of political/economic organizations in your community?
   ①A few ②Less the average ③Average ④More than average ⑤A lot
10. How many of the political/economic organizations represent your interests?
   ①A few ②Less the average ③Average ④More than average ⑤A lot
11. How many of these groups and organizations possess broad social connections?
   ①A few ②Less the average ③Average ④More than average ⑤A lot
12. How many of the political/economic organizations will help you upon your request?
   ①A few ②Less the average ③Average ④More than average ⑤A lot

13. How do you rate the number of creational/cultural organizations in your community?

①A few ②Less the average ③Average ④More than average ⑤A lot

14. How many of the creational/cultural organizations represent your interests?

①A few ②Less the average ③Average ④More than average ⑤A lot

15. How many of the creational/cultural organizations possess broad social connections?

①A few ②Less the average ③Average ④More than average ⑤A lot

16. How many of the creational/cultural organizations will help you upon your request?

①A few ②Less the average ③Average ④More than average ⑤A lot

个人社会资本量表

1. 您有多少朋友？ E1 □

   ①没有　②有　③一半　④较多　⑤全部

2. 您有多少同学？ E2 □

   ①没有　②有　③一半　④较多　⑤全部

3. 在您的所有朋友、同学中，有多少能够完全信任？ E3 □

   ①没有　②有　③一半　④较多　⑤全部

4. 在您的所有亲人中，有多少能够完全信任？ E4 □

   ①没有　②有　③一半　④较多　⑤全部

5. 在您的所有亲人、邻居、朋友、同事和同学中，有多少社会交往非常广泛？ E5 □

   ①没有　②有　③一半　④较多　⑤全部

6. 在您的所有亲人、邻居、朋友、同事和同学中，有多少有正式的工作？ E6 □

   ①没有　②有　③一半　④较多　⑤全部

7. 如果您有要求，有多少同事、同学一定会帮忙？ E7 □

   ①没有　②有　③一半　④较多　⑤全部

8. 如果您有要求，有多少朋友一定会帮忙？ E8□

   ①没有　②有　③一半　④较多　⑤全部

9. 在您的社区里面，有多少文化、娱乐和休闲组织？ E9□

   ①没有　②有　③一半　④较多　⑤全部

10. 这些文化、娱乐和休闲组织，你对其中多少感兴趣？ E10□

    ①没有　②有　③一半　④较多　⑤全部

11. 如果您有要求，这些文化、娱乐和休闲组织有多少会对您提供帮助？ E11□

    ①没有　②有　③一半　④较多　⑤全部

12. 在您的社区里面，有多少政府、政治、经济和文化组织？ E12□

    ①没有　②有　③一半　④较多　⑤全部

13. 这些政府、政治、经济和文化组织，你对其中多少感兴趣？ E13□

    ①没有　②有　③一半　④较多　⑤全部

14. 如果您有要求，这些政府、政治、经济和文化组织有多少会对您提供帮助？ E14□

①没有 ②有 ③一半 ④较多 ⑤全部

15. 上述这些组织中，有多少具有广泛的社会联系？ E15□

①没有 ②有 ③一半 ④较多 ⑤全部

16. 上述这些组织中，有多少具有广泛的社会影响力？ E16□

①没有 ②有 ③一半 ④较多 ⑤全部
